# Dose escalation and pharmacokinetic study of a humanized anti-HER2 monoclonal antibody in patients with HER2/*neu*-overexpressing metastatic breast cancer

**DOI:** 10.1038/sj.bjc.6690343

**Published:** 1999-12

**Authors:** Y Tokuda, T Watanabe, Y Omuro, M Ando, N Katsumata, A Okumura, M Ohta, H Fujii, Y Sasaki, T Niwa

**Affiliations:** Department of Surgery, Tokai University School of Medicine, Bohseidai, Isehara, Kanagawa 259-1193, Japan; National Cancer Center Hospital, Tokyo 104-0045, Japan; National Cancer Center Hospital East, Kashiwa, Chiba 277-0882, Japan; Mitsubishi Chemical Corporation, Yokohama Research Center, Kanagawa 227–8502, Japan

**Keywords:** HER2/*neu*, humanized monoclonal antibody, pharmacokinetics, phase I study

## Abstract

We conducted a phase I pharmacokinetic dose escalation study of a recombinant humanized anti-p185^HER2^ monoclonal antibody (MKC-454) in 18 patients with metastatic breast cancer refractory to chemotherapy. Three or six patients at each dose level received 1, 2, 4 and 8 mg kg^–1^ of MKC-454 as 90-min intravenous infusions. The first dose was followed in 3 weeks by nine weekly doses. Target trough serum concentration has been set at 10 μg ml^–1^ based on in vitro observations. The mean value of minimum trough serum concentrations at each dose level were 3.58 ± 0.63, 6.53 ± 5.26, 40.2 ± 7.12 and 87.9 ± 23.5 μg ml^–1^ respectively. At 2 mg kg^–1^, although minimum trough serum concentrations were lower than the target trough concentration with a wide range of variation, trough concentrations increased and exceeded the target concentration, as administrations were repeated weekly. Finally 2 mg kg^–1^ was considered to be sufficient to achieve the target trough concentration by the weekly dosing regimen. One patient receiving 1 mg kg^–1^ had grade 3 fever, one at the 1 mg kg^–1^ level had severe fatigue defined as grade 3, and one at 8 mg kg^–1^ had severe bone pain of grade 3. No antibodies against MKC-454 were detected in any patients. Objective tumour responses were observed in two patients; one receiving 4 mg kg^–1^ had a partial response in lung metastases and the other receiving 8 mg kg^–1^ had a complete response in soft tissue metastases. These results indicate that MKC-454 is well tolerated and effective in patients with refractory metastatic breast cancers overexpressing the HER2 proto-oncogene. Further evaluation of this agent with 2–4 mg kg^–1^ weekly intravenous infusion is warranted. © 1999 Cancer Research Campaign


*British Journal of Cancer* (1999) **81**(8), 1419–1425
© 1999 Cancer Research Campaign
Article no. bjoc.1999.0861

### Dose escalation and pharmacokinetic study of a humanized anti-HER2 monoclonal antibody in patients with HER2/*neu*-overexpressing metastatic breast cancer

**Y Tokuda[1], T Watanabe[2], Y Omuro[2], M Ando[2], N Katsumata[2], A Okumura[1], M Ohta[1], H Fujii[3], Y Sasaki[3], T Niwa[4] and T Tajima[1]**

[1]Department of Surgery, Tokai University School of Medicine, Bohseidai, Isehara, Kanagawa 259-1193, Japan; [2]National Cancer Center Hospital, Tokyo 104-0045, Japan; [3]National Cancer Center Hospital East, Kashiwa, Chiba 277-0882, Japan; [4]Mitsubishi Chemical Corporation, Yokohama Research Center, Kanagawa 227–8502, Japan

**Summary** We conducted a phase I pharmacokinetic dose escalation study of a recombinant humanized anti-p185$^{HER2}$ monoclonal antibody (MKC-454) in 18 patients with metastatic breast cancer refractory to chemotherapy. Three or six patients at each dose level received 1, 2, 4 and 8 mg kg$^{-1}$ of MKC-454 as 90-min intravenous infusions. The first dose was followed in 3 weeks by nine weekly doses. Target trough serum concentration has been set at 10 μg ml$^{-1}$ based on in vitro observations. The mean value of minimum trough serum concentrations at each dose level were 3.58 ± 0.63, 6.53 ± 5.26, 40.2 ± 7.12 and 87.9 ± 23.5 μg ml$^{-1}$ respectively. At 2 mg kg$^{-1}$, although minimum trough serum concentrations were lower than the target trough concentration with a wide range of variation, trough concentrations increased and exceeded the target concentration, as administrations were repeated weekly. Finally 2 mg kg$^{-1}$ was considered to be sufficient to achieve the target trough concentration by the weekly dosing regimen. One patient receiving 1 mg kg$^{-1}$ had grade 3 fever, one at the 1 mg kg$^{-1}$ level had severe fatigue defined as grade 3, and one at 8 mg kg$^{-1}$ had severe bone pain of grade 3. No antibodies against MKC-454 were detected in any patients. Objective tumour responses were observed in two patients; one receiving 4 mg kg$^{-1}$ had a partial response in lung metastases and the other receiving 8 mg kg$^{-1}$ had a complete response in soft tissue metastases. These results indicate that MKC-454 is well tolerated and effective in patients with refractory metastatic breast cancers overexpressing the HER2 proto-oncogene. Further evaluation of this agent with 2–4 mg kg$^{-1}$ weekly intravenous infusion is warranted. © 1999 Cancer Research Campaign

**Keywords:** HER2/*neu*; humanized monoclonal antibody; pharmacokinetics; phase I study

The c-*erbB*-2/HER2 proto-oncogene encodes a receptor-type tyrosine kinase (Yarden and Ullrich, 1988) corresponding to, but distinct from, the epidermal growth factor receptor (Coussens et al, 1985; Yamamoto et al, 1986). The HER2 product consists of three domains, extracellular, transmembrane and intracellular domains which has tyrosine kinase activity making the HER2 product autophosphorylated. Appreciable amplification and/or overexpression of this gene has been demonstrated in a variety of adenocarcinomas including breast cancer (King et al, 1985; Slamon et al, 1989). However, the expression of this gene in normal adult tissues is weak (De Potter et al, 1989; Press et al, 1990). Therefore, the HER2 product is thought to be a useful target for antibody therapy of cancers overexpressing the HER2 gene.

Several studies of murine monoclonal antibodies (Mabs) directed against the HER2 product have already been reported to have in vitro and in vivo anti-tumour effects (Drebin et al, 1985; Hudziak et al, 1989; Hancock et al, 1991; Stancovski et al, 1991; Tagliabue et al, 1991; Harwerth et al, 1992; Kasprzyk et al, 1992). However, human anti-mouse antibody response during therapy would be a major limitation in the clinical application of such murine mabs (Schroff et al, 1985; Shawler et al, 1985). Carter and his colleagues constructed a humanized antibody containing only the antigen-binding loops from a murine mab against the extracellular domain of the HER2 gene product and human variable region framework residues plus IgG1 constant domains (Carter et al, 1992). A clinical study of the humanized antibody revealed some objective responses without antibodies against it (Baselga et al, 1996).

We report here the results of a phase I dose escalation study of the humanized mab (MKC-454) against the HER2 product in patients with metastatic breast cancer refractory to conventional chemotherapeutic agents and positive for this oncogene product.

*Received 20 November 1998*
*Revised 14 May 1999*
*Accepted 7 June 1999*

*Correspondence to:* Y Tokuda

### PATIENTS AND METHODS

### Patient eligibility

Patients with metastatic breast cancer refractory to conventional chemo or endocrine therapies and overexpressing the HER2 product were eligible for this study. Patients were considered refractory to endocrine therapy if hormone receptors were negative or tumour growth was noted during treatment with tamoxifen, and were considered refractory to chemotherapy if disease progression was noted after treatment with doxorubicin-containing regimens. All patients had measurable disease,

**Table 1** Patient demographics

| Parameter | No. |
|---|---|
| No. of patients | 18 |
| Age median (years) (range) | 51 (32–64) |
| Performance score (ECOG) | |
| 0 | 10 |
| 1 | 7 |
| 2 | 1 |
| Receptor status | |
| Oestrogen receptor | |
| Positive | 3 |
| Negative | 11 |
| Unknown | 4 |
| Progesterone receptor | |
| Positive | 3 |
| Negative | 10 |
| Unknown | 5 |
| Histological grade | |
| II | 3 |
| III | 12 |
| Unknown | 3 |
| Prior treatment | |
| Chemotherapy | |
| Adjuvant chemotherapy | 12 |
| Neoadjuvant chemotherapy | 1 |
| Metastatic disease (no. of regimens) | |
| 1 | 7 |
| 2 | 7 |
| >2 | 4 |
| Median (range) | 2 (1–4) |
| Hormonal therapy | |
| Adjuvant therapy | 11 |
| Metastatic disease | 6 |

satisfactory haematologic, hepatic, renal and pulmonary function with an Eastern Cooperative Oncology Group (ECOG) performance status grade 3 or less. Patients who had chemotherapy within 4 weeks (6 weeks for mitomycin or nitrosurea) or hormonal therapy within 2 weeks before study entry were ineligible. Written informed consent was mandatory before study entry.

The expression of HER2 was determined by immunohistochemical staining of the paraffin-embedded thin sections of the primary or metastatic tumours using rabbit polyclonal antibodies (DAKO, Glostrup, Denmark and NICHIREI, Tokyo, Japan) or a murine monoclonal antibody (Lab Corp, North Carolina, USA) against the human HER2 product. Tumours were considered to overexpress HER2 if at least 10% of tumour cells had positive membrane staining.

### Treatment

A recombinant humanized anti-HER2 mAb (trastuzumab, MKC-454) was constructed by molecular engineering from a murine mAb recognizing the extracellular domain of the HER2 product (Carter *et al*, 1992). The drug was administered intravenously at a constant infusion rate for 90 min. The starting dose level of MKC-454 was 1 mg $kg^{-1}$, and subsequent dose escalations were to 2, 4 and 8 mg $kg^{-1}$. The first dose was followed in 3 weeks by nine weekly doses. A minimum of three patients assessable for toxicity were treated at each dose level. If one of the first three patients entered at any dose level experienced a dose-limiting toxicity, an additional three patients were entered at that dose level. Toxicities were graded according to the JCOG toxicity criteria (Tobinai et al, 1993). Dose-limiting toxicity was defined as any grade 3 or 4 toxicity.

### Pharmacokinetics, determination of antibodies against the humanized antibody and circulating shed antigen

Serum concentrations of MKC-454 were determined in a receptor binding assay that detects binding with the extracellular domain of the HER2 product. The recombinant extracellular domain of the HER2 product provided from Genentech, Inc. was coated on 96-well microtitre plates. MKC-454 bound to the coated antigen was detected by horseradish peroxidase-labelled goat anti-human IgG Fc. The quantitative limit for MKC-454 in serum samples was 156 ng $ml^{-1}$. The presence of antibodies against MKC-454 was determined with a bridging-type titre enzyme-linked immunosorbent assay (ELISA). Serum concentrations of circulating extracellular domain of the HER2 product, circulating shed antigen were measured using an ELISA. A pair of polyclonal antibodies against extracellular domain of the HER2 product were provided from Genentech, Inc. The quantitative limit for circulating shed antigen was 1.54 ng $ml^{-1}$. Pharmacokinetic parameters of MKC-454 after first administration were determined for each patient. Maximum serum concentration ($C_{max}$) was the observed value.

Half-life ($t_{1/2}$), area under concentration-time curve ($AUC_{0-\infty}$), distribution volume (V) and serum clearance (CL) were estimated by non-compartmental model of WinNonlin Standard Japanese Edition Version 1.1 (Scientific Consulting Inc.). As for the trough serum levels during the weekly treatment period, minimum and maximum values were obtained for each patient.

### Evaluation of response

All responses were reviewed centrally by an independent extramural evaluation committee. A complete response (CR) was defined as the complete resolution of all measurable tumours for a duration of at least 4 weeks. Partial response (PR) was defined as a ≥ 50% reduction in the sum of the products of the two longest perpendicular diameters of all measurable tumours for a duration of at least 4 weeks, a minimal response (MR) as a ≥ 25% but less than 50% reduction in the sum of the products of the two longest perpendicular diameters of all tumours, and progressive disease (PD) as a ≥ 25% increase in any measurable lesions or the appearance of any new lesion.

### RESULTS

Patient characteristics are listed in Table 1. All patients had received chemotherapy for metastatic disease and 11 patients had received two or more regimens. All of the metastatic diseases had become refractory to anthracycline-containing regimens. Although hormonal therapy for metastatic disease was also given to a third of the patients, 11 primary tumours were negative for oestrogen receptor. Twelve patients had pathological grade III primary tumours.

Serum concentration-time profiles after first administration are illustrated in Figure 1 and estimated pharmacokinetic parameters for each patient are summarized in Table 2. Mean $C_{max}$ increased in good proportion to dose. Mean distribution volume (V) ranging from 52.1 to 74.3 ml kg, was independent of dose, and approximated the serum space. Half-life ($t_{1/2}$) increased from 2.7 days at

 *© 1999 Cancer Research Campaign*

**Table 2** Single dose pharmacokinetic parameters of MKC-454 in each patient

| Dose (mg kg$^{-1}$) | Patient no. | $C_{max}$ (ng ml$^{-1}$) | $t_{1/2}$ (day) | $AUC_{0-\infty}$ (µg day ml$^{-1}$) | V (ml kg$^{-1}$) | CL (ml day kg$^{-1}$) |
|---|---|---|---|---|---|---|
| 1 | 1–102 | 17 506 | 2.6 | 67 | 56.0 | 15.0 |
| | 1–103[a] | 14 994 | 2.3 | 47 | 45.2 | 14.2 |
| | 1–104 | 21 664 | 2.4 | 70 | 49.6 | 14.2 |
| | 1–105 | 22 230 | 3.2 | 94 | 48.0 | 10.6 |
| | 1–106 | 18 590 | 2.8 | 85 | 45.9 | 11.8 |
| | 3–107 | 15 770 | 2.3 | 53 | 61.1 | 18.9 |
| | Mean[b] | 19 152 | 2.7 | 74 | 52.1 | 14.1 |
| | s.d.[b] | 2 750 | 0.4 | 16 | 6.3 | 3.2 |
| 2 | 3–108 | 52 370 | 1.6 | 158 | 33.4 | 12.7 |
| | 2–109 | 42 500 | 2.3 | 198 | 49.1 | 10.1 |
| | 1–110 | 35 270 | 5.3 | 190 | 75.4 | 10.5 |
| | Mean | 43 413 | 3.1 | 182 | 52.6 | 11.1 |
| | s.d. | 8 537 | 2.0 | 21 | 21.2 | 1.4 |
| 4 | 3–111 | 62 340 | 10.2 | 549 | 85.9 | 7.3 |
| | 3–112 | 62 720 | 7.7 | 611 | 77.0 | 6.5 |
| | 1–113 | 92 320 | 8.4 | 746 | 60.0 | 5.4 |
| | Mean | 72 460 | 8.8 | 635 | 74.3 | 6.4 |
| | s.d. | 17 200 | 1.3 | 101 | 13.2 | 1.0 |
| 8 | 3–114 | 167 600 | 6.4 | 928 | 68.8 | 8.6 |
| | 1–115 | 193 500 | 15.4 | 2101 | 77.1 | 3.8 |
| | 1–116 | 197 050 | 10.3 | 1954 | 56.9 | 4.1 |
| | 2–117 | 149 850 | 10.2 | 1551 | 72.4 | 5.2 |
| | 3–118 | 176 700 | 11.3 | 1810 | 67.5 | 4.4 |
| | 3–119 | 133 250 | 9.0 | 1111 | 79.5 | 7.2 |
| | Mean | 169 658 | 10.4 | 1576 | 70.4 | 5.6 |
| | s.d. | 24 862 | 3.0 | 471 | 8.1 | 1.9 |

[a]0.67 mg/kg of dose was used for calculation of pharmacokinetic parameters because infusion was discontinued at 60 min.
[b]Patient no. 1–103 was deleted from calculation of mean and s.d. due to different dose.

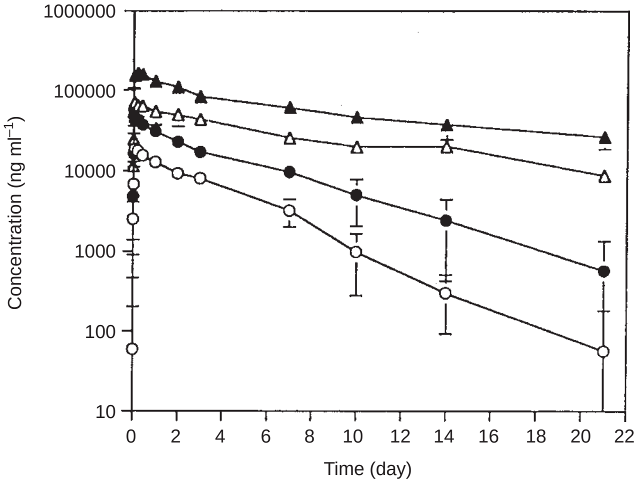

**Figure 1** Serum concentration-time profiles of MKC-454 after first administration. The values of serum concentration at a dose level of 1 mg kg$^{-1}$ (open circle), 2 mg kg$^{-1}$ (closed circle), 4 mg kg$^{-1}$ (open triangle), or 8 mg kg$^{-1}$ (closed triangle) represent mean ± s.d.

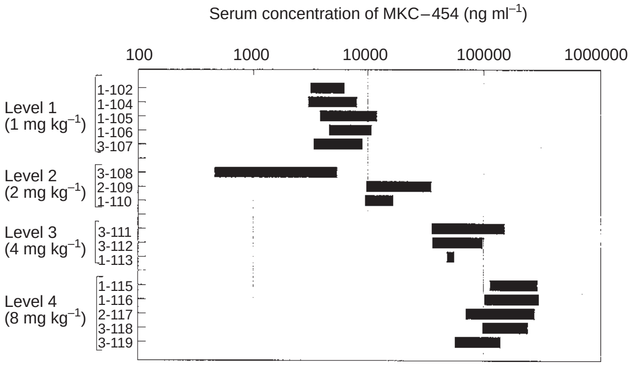

**Figure 2** Trough serum concentrations of MKC-454. Left and right ends of each horizontal bar represent minimum and maximum trough serum concentration of MKC-454 during weekly administration the lowest dose (1 mg kg$^{-1}$) to 10.4 days at the highest dose (8 mg kg$^{-1}$). Mean serum clearance (CL) decreased dose-dependently from 14.1 to 5.6 ml day$^{-1}$ kg$^{-1}$. Trough serum concentrations during repeated administration of MKC-454 are individually summarized in Figure 2. Maximum values of trough serum concentrations at each dose level were 9.19 ± 2.26, 19.1 ± 15.1, 102 ± 47.9, and 248 ± 64.4 µg ml$^{-1}$ respectively. The mean value of minimum trough serum concentrations at each dose level were 3.58 ± 0.63, 6.53 ± 5.26, 40.2 ± 7.12 and 87.9 ± 23.5 µg ml$^{-1}$ respectively. Figure 3 shows the serum concentrations of circulating extracellular domain of the HER2 product, shed antigen. One patient (Patient No. 3–108) at 2 mg kg$^{-1}$ with high values of circulating shed antigen showed reduced values of the trough serum concentrations compared with those of the other two

*© 1999 Cancer Research Campaign* 

**Table 3** Toxicity of MKC-454

| | Dose 1 mg $kg^{-1}$ $n = 6$ Grade | | | Dose 2 mg $kg^{-1}$ $n = 3$ Grade | | | Dose 4 mg $kg^{-1}$ $n = 3$ Grade | | | Dose 8 mg $kg^{-1}$ $n = 6$ Grade | | |
|---|---|---|---|---|---|---|---|---|---|---|---|---|
| | I | II | III | I | II | III | I | II | III | I | II | III |
| Fever | – | 2 | 1 | – | 1 | – | 1 | 1 | – | – | 1 | – |
| Nausea/vomiting | – | 1 | – | – | – | – | 1 | – | – | – | 1 | – |
| Gastrointestinal disorder | – | – | 1 | – | – | – | – | – | – | – | – | – |
| Liver | | | | | | | | | | | | |
| ASAT | 4 | – | – | – | – | – | – | 1 | – | 1 | – | – |
| ALAT | 2 | – | – | – | – | – | – | 1 | – | – | – | – |
| Tachycardia | – | – | – | – | – | – | – | 1 | – | 1 | – | – |
| Rigors | 1 | – | – | 1 | – | – | – | – | – | – | – | – |
| Feeling of warmth | 1 | – | – | 1 | – | – | – | – | – | – | – | – |
| Bone pain | – | – | – | – | – | – | – | – | – | – | – | 1 |
| Tumour pain | – | – | – | – | – | – | – | – | – | – | 1 | – |
| Cough | – | – | – | 1 | – | – | – | – | – | – | – | – |
| Oedema | – | – | – | 1 | – | – | – | – | – | – | – | – |
| Malaise | – | – | – | 1 | – | – | – | – | – | – | – | – |

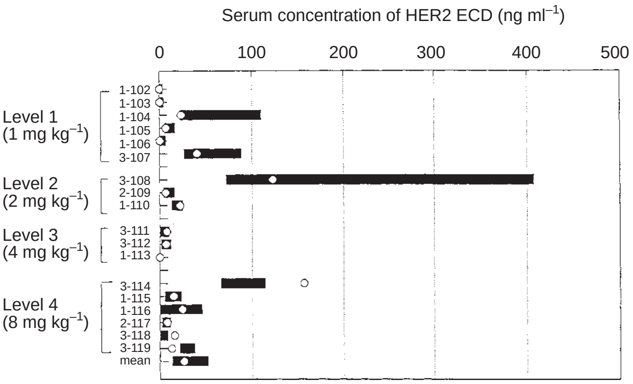

**Figure 3** Serum concentrations of circulating extracellular domain of HER2 product (HER2 ECD). Open circles represent concentrations of HER2 ECD prior to the commencement of the treatment. Left and right ends of each horizontal bar represent minimum and maximum concentrations of circulating HER2 ECD during weekly administration

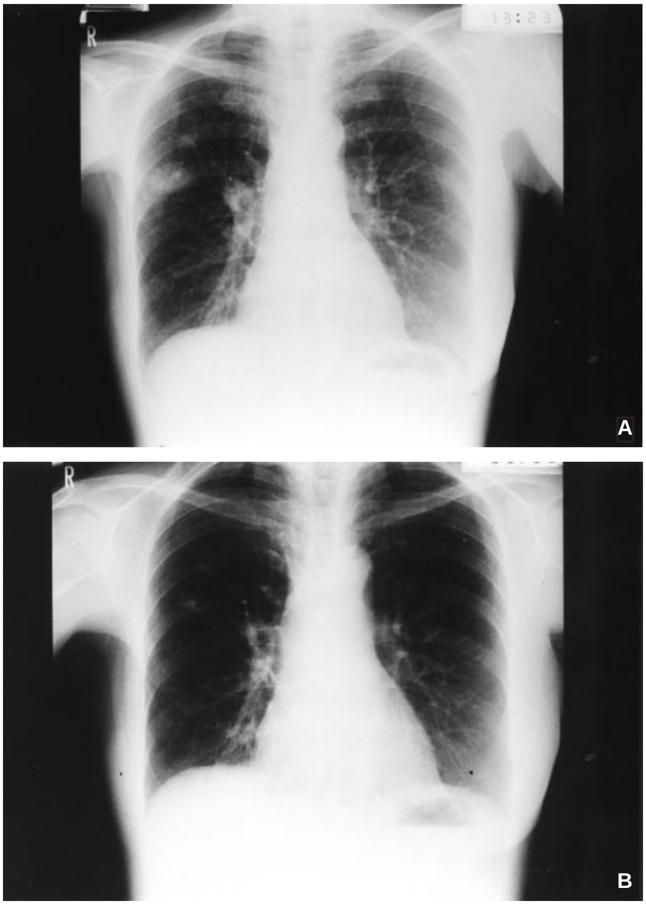

**Figure 4** Metastatic tumours in the lung before treatment with MKC-454 (**A**) and 7 months later showing marked response (**B**)

patients at the same dose level. Antibodies against MKC-454 were not detected in any patient.

From a total of 134 administrations of MKC-454, drug-related toxicity is shown in Table 3. A 38°C or higher fever was observed in six patients. One patient at 1 mg $kg^{-1}$ experienced grade 3 fever up to 40.7°C while receiving the first administration. Despite the temporary event, further treatment with MKC-454 was not given. Although one patient had no bone metastasis, the patient at the dose of 8 mg $kg^{-1}$ experienced severe generalized bone pain judged as grade 3. Although the pain subsided within 8 h, administration of MKC-454 was discontinued.

After the clinical trial had been completed, treatment responses of all the 18 patients were assessed by the committee confirming toxicity and efficacy. Data are summarized in Table 4. Although no response was observed at the dose level of 1 mg $kg^{-1}$, one patient with cervical and mediastinal lymph node metastases had a MR at the dose level of 2 mg $kg^{-1}$. One patient with multiple lung metastases had a PR at 4 mg $kg^{-1}$. Due to her good response to date, she has received continuous weekly administration of MKC-454 4 mg $kg^{-1}$ for 18 months (Figure 4). A patient at 8 mg $kg^{-1}$ with skin and bilateral supraclavicular lymph node metastases had a CR for 3 months (Figure 5). However, skin metastasis relapsed

 *© 1999 Cancer Research Campaign*

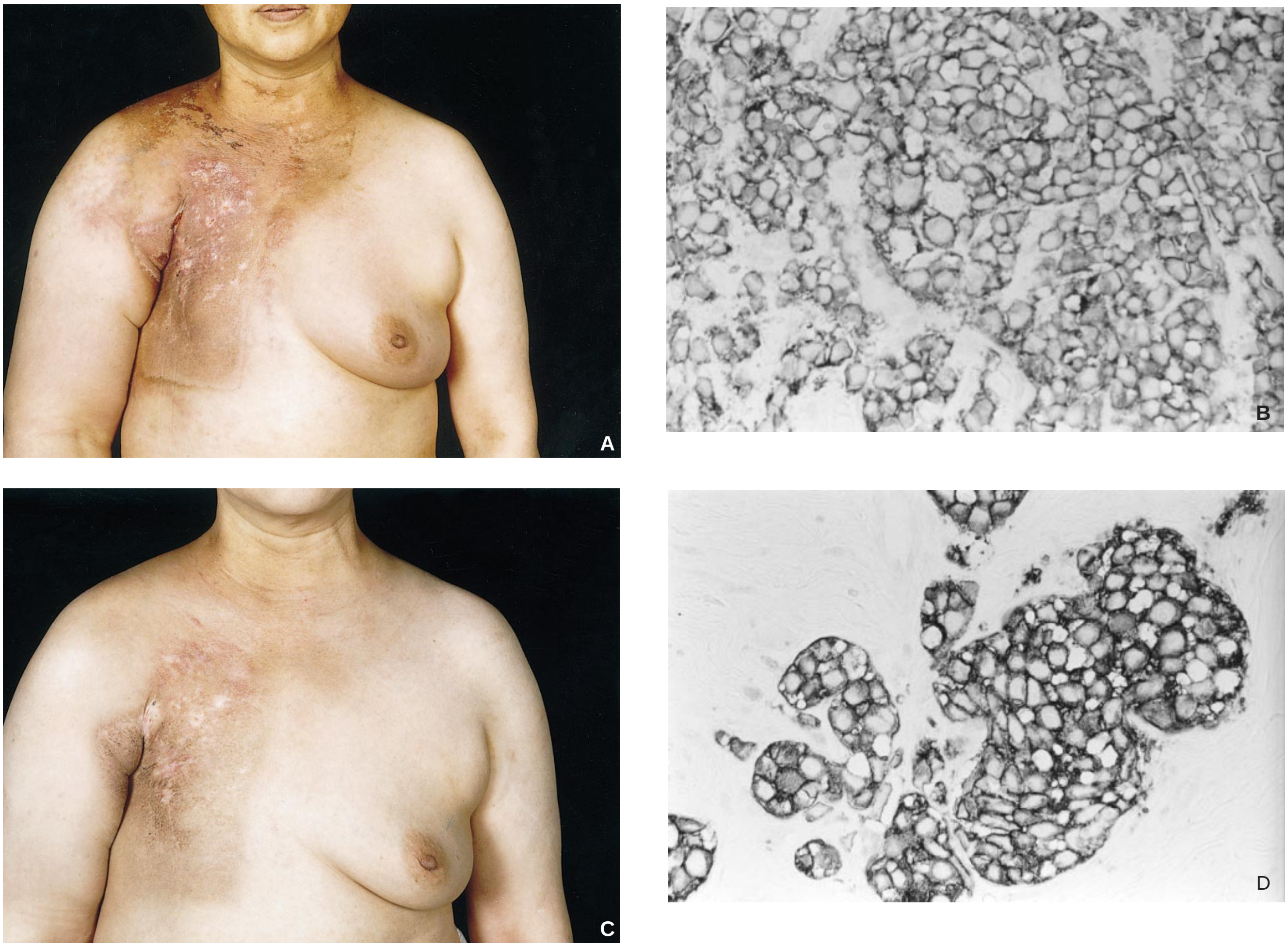

**Figure 5** Skin metastasis before treatment with MKC-454 (**A**) showing strong positivity for HER2 product (**B**) and 4 months later showing pathologically complete response (**C**). Relapsing tumour cells with positive HER2 product (**D**)

**Table 4** Response to MKC-454

| Dose mg kg$^{-1}$ | NE | PD | NC | MR | PR | CR | Total |
|---|---|---|---|---|---|---|---|
| 1 | 1 | 4 | 1 | – | – | – | 6 |
| 2 | – | 2 | – | 1 | – | – | 3 |
| 4 | – | 2 | – | – | 1 | – | 3 |
| 8 | 1 | 1 | 1 | 2 | – | 1 | 6 |
| Total | 2 | 9 | 2 | 3 | 1 | 1 | 18 |

NE, not evaluable; PD, progressive disease; NC, no change; MR, minimal response; PR, partial response; CR, complete response.

during the course of the treatment. It is of some interest that the relapsed tumour cells were still strongly overexpressing the HER2 product. Her circulating shed antigen had been found low through the entire treatment course. Therefore it was considered that the circulating shed antigen had not affected her MKC-454 pharmacokinetics.

### DISCUSSION

The HER2 product is thought to be a unique and useful target for antibody therapy of cancers overexpressing the HER gene. Several series of murine mAbs directed against the extracellular domain of the HER2 gene product have been developed and examined to reveal their anti-tumour effects, mainly in vitro. However, human anti-mouse antibody response during the therapy would be a major limitation on the clinical application of such murine mAbs. Preclinical studies suggested that a humanized antibody would have the same or greater anti-tumour potency in clinical trials than its murine counterpart (Tokuda et al, 1996). Therefore, a humanized antibody against the HER2 gene product is expected to be useful in the clinic.

Baselga and his colleagues reported the first clinical evidence of the anti-tumour activity of a humanized antibody against the HER2 product (Baselga et al, 1996). They set 10 µg ml$^{-1}$ as the target trough serum concentration based on in vitro data (Carter et al, 1992). Our preclinical data also indicated 10 µg ml$^{-1}$ as a minimally effective concentration for anti-proliferation effects and antibody-dependent cell-mediated cytotoxicity (ADCC) (Tokuda et al, 1996). The dosage and schedule of the antibody administration in this clinical trial was based on unpublished phase I trials in the United States. In this clinical trial, non-linear pharmacokinetics of MCK-454 was demonstrated as previously described for other monoclonal antibodies (Koizumi et al, 1986; Eger et al, 1987). According to our data, 4 mg kg$^{-1}$ is sufficient to achieve the target

*© 1999 Cancer Research Campaign* 

trough serum concentration (10 μg ml$^{-1}$). At 2 mg kg$^{-1}$, although minimum trough serum concentrations were lower than the target concentration with a wide range of variation, trough serum concentrations increased and exceeded the target concentration in the case of patients with absent or low circulating shed antigen (Patient nos 2–109 and 1–110), as administrations were repeated weekly. The maximum trough serum concentration of patient nos 2–109 and 1–110 were 35.4 and 16.7 μg ml$^{-1}$ respectively. Although 16.7 μg ml$^{-1}$ was lower due to discontinuation of treatment before reaching steady state, these values corresponded to 54 ± 32 μg ml$^{-1}$ observed in the previous clinical trial (Pegram et al, 1998) which used 100 mg per body as weekly dose level. These suggested that 2 mg kg$^{-1}$ was also sufficient to achieve the target concentration by the weekly dosing regimen. A patient with high circulating shed antigen at 2 mg kg$^{-1}$ (Patient no. 3–108) showed reduced serum concentrations. This observation was consistent with previous reports suggesting that high circulating shed antigen decrease the half-life and the trough serum concentrations of humanized mAb against HER2 product (Baselga et al, 1996; Pegram et al, 1998). Therefore, monitoring of circulating shed antigen level is considered to be essential in MKC-454 therapy and more than 2 mg kg$^{-1}$ may be necessary in patients with high circulating shed antigen.

Responding cases, including MRs, were observed at the dose levels of 2 mg kg$^{-1}$ or more. One patient with a PR for lung metastases who relapsed after high-dose chemotherapy received 4 mg kg$^{-1}$ of MKC-454. The other responding patient receiving 8 mg kg$^{-1}$ achieved a CR for skin metastases, which had been heavily treated with chemotherapy and radiotherapy. Despite continuous treatment of MKC-454, it is noteworthy that the HER2 product was still remarkably overexpressed in tumour cells of relapsed skin lesions. Circulating shed antigen in this patient had been low. It is possible that tumour clones overexpressing truncated forms of the extracellular domain of the HER2 product appeared (Scott et al, 1993), but confirmation of this would require further analysis.

Toxicity caused by the treatment was well tolerated compared with that of chemotherapeutic drugs. Two patients discontinued the treatment after the first administration. However, both toxicities were temporary and could have been prevented with prophylactic use of drugs such as anti-inflammatory agents, which was prohibited in the protocol of this study.

In conclusion, MKC-454 showed clinical activity with minimum toxicity. These findings indicate that this treatment deserves further clinical trials at suggested weekly intravenous infusions of 2–4 mg kg$^{-1}$.

### ACKNOWLEDGEMENTS

We thank Ms Rieko Matsumoto, Dr Kazuhiro Takahashi and Dr Jindow Itoh of the Mitsubishi Chemical Corporation for their helpful advice and cooperation. We also thank Dr Robert L Cohen of the Molecular Oncology Department and Dr Sharon A Baughman of the Pharmacokinetics, Genentec, Inc, both for helpful comments during the preparation of this manuscript. This work was supported in part by Grant-in-Aids from the Ministry of Education, Science and Culture, and from the Ministry of Health and Welfare of Japan.

### REFERENCES

Baselga J, Tripathy D, Mendelsohn J, Baughman S, Benz CC, Danits L, Sklarin NT, Seidman AD, Hudis CA, Moore J, Rosen PP, Twaddell T, Henderson IC and Norton L (1996) Phase II study of weekly intravenous recombinant humanized anti-p185HER2 monoclonal antibody in patients with HER2/neu-overexpressing metastatic breast cancer. *J Clin Oncol* **14**: 737–744

Carter P, Presta L, Gorman CM, Ridgway JBB, Henner D, Wong WLT, Rowland AM, Kotts C, Carver ME and Shepard HM (1992) Humanization of an anti-p185HER2 antibody for human cancer therapy. *Proc Natl Acad Sci USA* **89**: 4285–4289

Coussens L, Yang-Feng TL, Liao Y-C, Chen E, Gray A, McGrath J, Seeburg PH, Liebermann TW, Schlessinger J, Franke U, Levison A and Ullrich A (1985) Tyrosine kinase receptor with extensive homology to EGF receptor shares chromosomal location with neu oncogene. *Science* **230**: 1132–1139

De Potter CR, Van Daele S, Van De Vijver MJ, Pauwels C, Maertens G, De Boever J, Vandekerckhove D and Roels H (1989) The expression of the neu oncogene product in breast lesions and in normal fetal and adult human tissues. *Histopathology* **15**: 315–326

Drebin JA, Link VC, Stern DF, Weinberg RA and Green MI (1985) Down modulation of an oncogene protein product and reversion of the transformed phenotype by monoclonal antibodies. *Cell* **41**: 695–706

Eger RR, Covell DG, Carrasquillo JA, Abrams PG, Foon KA, Reynolds JC, Schroff RW, Morgan AC, Larson SM and Weinstein JN (1987) Kinetic model for the biodistribution of an $^{111}$In-labeled monoclonal antibody in humans. *Cancer Res* **47**: 3328–3336

Hancock MC, Langton BC, Chan T, Toy P, Monahan JJ, Mischak RP and Shawver LK (1991) A monoclonal antibody against the c-*erb*B-2 protein enhances the cytotoxicity of *cis*-diamminedichloroplatinum against human breast and ovarian tumour cell lines. *Cancer Res* **51**: 4575–4580

Harwerth I-M, Wels W, Marte BM and Hynes NE (1992) Monoclonal antibodies against the extracellular domain of the *erb*B-2 receptor function as partial ligand agonists. *J Biol Chem* **267**: 15160–15167

Hudziak RM, Lewis GD, Winget M, Fendly BM, Shepard M and Ullrich A (1989) p185HER2 monoclonal antibody has antiproliferative effects in vitro and sensitized human breast tumor cells to tumor necrosis factor. *Mol Cell Biol* **9**: 1165–1172

Kasprzyk PG, Song SU, Di Fiore PP and King CR (1992) Therapy of an animal model of human gastric cancer using a combination of anti-*erb*B-2 monoclonal antibodies. *Cancer Res* **52**: 2771–2776

King CR, Kraus MH and Aronson SA (1985) Amplification of a novel v-*erb*B-related gene in a human mammary carcinoma. *Science* **229**: 974–976

Koizumi K, DeNardo GL, DeNardo SJ, Hays MT, Hines HH, Scheibe PO, Peng J, Macey DJ, Tonami N and Hisada K (1986) Multicompartmental analysis of the kinetics of radioiodinated monoclonal antibody in patients with cancer. *J Nucl Med* **27**: 1243–1254

Pegram MD, Lipton A, Hayes DF, Weber BL, Baselga JM, Tripathy D, Baly D, Baughman SA, Twaddell T, Glaspy JA and Slamon DJ (1998) Phase II study of receptor-enhanced chemosensitivity using recombinant humanized anti-p185HER2/*neu* monoclonal antibody plus cisplatin in patients with HER2/*neu*-overexpressing metastatic breast cancer refractory to chemotherapy treatment. *J Clin Oncol* **16**: 2659–2671

Press MF, Cerdon-Cardo C and Slamon DJ (1990) Expression of the HER-2/*neu* proto-oncogene in normal human adult and fetal tissues. *Oncogene* **5**: 953–962

Schroff RW, Foon KA, Beatty SM, Oldham RK and Morgan Jr AC (1985) Human anti-murine immunoglobulin responses in patients receiving monoclonal antibody therapy. *Cancer Res* **45**: 879–885

Scott GK, Robles R, Park JW, Montgomery PA, Daniel J, Holmes WE, Lee J, Keller GA, Li WL, Fendly BM, Wood WI, Shepard HM and Benz CC (1993) A truncated intracellular HER2/neu receptor produced by alternative RNA processing affects growth of human carcinoma cells. *Mol Cell Biol* **13**: 2247–2257

Shawler DL, Bartholomew RM, Smith LM and Dillman RO (1985) Human immune response to multiple injection of murine monoclonal IgG. *J Immunol* **135**: 1530–1535

Slamon DJ, Godolphin W, Jones LA, Holt JA, Wong SG, Keith DE, Levin WJ, Stuart SG, Udove J, Ullrich A and Press MF (1989) Studies of the HER-2/neu proto-oncogene in human breast and ovarian cancer. *Science* **244**: 707–712

Stankovski I, Hurwitz E, Leitner O, Ullrich A, Yarden Y and Sela M (1991) Mechanistic aspects of the opposing effects of monoclonal antibodies to the ERBB-2 receptor on tumor growth. *Proc Natl Acad Sci USA* **88**: 8691–8695

Tagliabue E, Centis F, Campiglio M, Mastroianni A, Martignone S, Pellegrini R, Casalini P, Lanzi C, Menard S and Colnaghi MI (1991) Selection of monoclonal antibodies which induce internalization and phosphorylation of p185HER2 and growth inhibition of cells with HER2/*neu* gene amplification. *Int J Cancer* **47**: 933–937

Tobinai K, Kohno A, Shimada Y, Watanabe T, Tamura T, Takeyama K, Narabayashi M, Fukutomi T, Kondo H, Shimoyama M and Suemasu K (1993) Toxicity

 *© 1999 Cancer Research Campaign*

grading criteria of the Japan Clinical Oncology Group. *Jpn J Clin Oncol* **23**: 250–257

Tokuda Y, Ohnishi Y, Shimamura K, Iwasawa M, Yoshimura M, Ueyama Y, Tamaoki N, Tajima T and Mitomi T (1996) In vitro and in vivo anti-tumour effects of a humanised monoclonal antibody against c-*erb*B-2 product. *Br J Cancer* **73**: 1362–1365

Yamamoto T, Ikawa S, Akiyama T, Semba K, Nomura N, Miyajima N, Saito T and Toyoshima K (1986) Similarity of protein encoded by human c-*erb*B-2 gene to epidermal growth factor receptor. *Nature* **319**: 230–234

Yarden Y and Ullrich A (1988) Growth factor receptor tyrosine kinases. *Annu Rev Biochem* **57**: 443–478

© 1999 Cancer Research Campaign
